# Single point mutations in global regulatory genes restore cephalosporin resistance in a low-MIC *Enterococcus faecium* natural isolate

**DOI:** 10.1128/aac.01948-25

**Published:** 2026-03-23

**Authors:** Miquel Sánchez-Osuna, Paula Bierge, Inmaculada Gómez-Sánchez, Judith Guitart-Matas, Víctor Monsálvez, Patricia Rabanal, Ana P. Pereira, Ana R. Freitas, Luisa Peixe, Mateu Espasa, Oriol Gasch, Carla Novais, Oscar Q. Pich

**Affiliations:** 1 Laboratori de Recerca en Microbiologia i Malalties Infeccioses, Hospital Universitari Parc Taulí, Institut d'Investigació i Innovació Parc Taulí (I3PT-CERCA), Universitat Autònoma de Barcelona, Sabadell, Spain; 2 Institut de Biotecnologia i Biomedicina, Universitat Autònoma de Barcelona, Bellaterra, Spain; 3 Servei de Malalties Infeccioses, Hospital Universitari Parc Taulí, Institut d’Investigació i Innovació Parc Taulí (I3PT-CERCA), Universitat Autònoma de Barcelona, Sabadell, Spain; 4 Unidade de Ciências Biomoleculares Aplicadas (UCIBIO), Faculdade de Farmácia, Universidade do Porto 26706, Porto, Portugal; 5 Laboratório Associado i4HB–Instituto para a Saúde e Bioeconomia, Faculdade de Farmácia, Universidade do Porto 26706, Porto, Portugal; 6 Unidade de Ciências Biomoleculares Aplicadas (UCIBIO), Instituto Universitário de Ciências da Saúde (1H-TOXRUN, IUCS-CESPU), Gandra, Portugal; 7 Servei de Microbiologia, Hospital Universitari Parc Taulí, Institut d’Investigació i Innovació Parc Taulí (I3PT-CERCA), Universitat Autònoma de Barcelona, Sabadell, Spain; The Peter Doherty Institute for Infection and Immunity, Melbourne, Victoria, Australia

**Keywords:** ampicillin-susceptible *Enterococcus faecium*, cephalosporin intrinsic resistance, CroS, NusG, RpoB, PBP5

## Abstract

*Enterococcus faecium* exhibits intrinsic resistance to cephalosporins (CPHs), yet the genetic determinants of this phenotype remain incompletely understood. To date, *E. faecium* strains with low minimum inhibitory concentrations (MICs) of CPH have only been described following genetic manipulation. At Parc Taulí University Hospital, we identified a clinical isolate of ampicillin-susceptible *E. faecium* (Efm5) that exhibited unusually low MICs to cefotaxime (1 mg/L), ceftriaxone (3 mg/L), and ceftaroline (0.19 mg/L). Upon single exposure to ceftriaxone (100 mg/L), Efm5 rapidly yielded variants with markedly increased MICs of ceftriaxone (>256 mg/L) and cefotaxime (>32 mg/L), while MICs of ampicillin and ceftaroline were unaffected. Whole-genome sequencing revealed that the high-MIC variants carried single-nucleotide polymorphisms leading to non-synonymous mutations in *croS*, *nusG*, or *rpoB* genes. Phenotypic assays confirmed that these mutations were associated with ceftriaxone resistance, and immunoblots revealed increased expression of penicillin-binding protein 5 (PBP5) in all the high-MIC variants. Transcriptional profiling showed upregulation of the *pbp5* operon, which includes *ftsW*, *psr*, and *pbp5*, in the *croS* variants. This study provides evidence that *E. faecium* isolates with low MICs to CPH can arise in clinical settings without laboratory manipulation, enabling further investigation of resistance pathways.

## INTRODUCTION

*Enterococcus faecium* is increasingly recognized for its reduced susceptibility to a wide range of commonly used antimicrobial agents, presenting a growing challenge for clinicians in managing infections caused by this pathogen (1). While β-lactams continue to serve as the primary therapeutic agents for susceptible gram-positive bacteria, *E. faecium* exhibits intrinsic resistance to cephalosporins (CPHs), complicating treatment strategies and limiting effective therapeutic options (2). Despite ongoing research, the precise molecular underpinnings of CPH resistance in *E. faecium* remain poorly understood.

CPH resistance in *E. faecium* is largely driven by low-affinity penicillin-binding proteins (PBPs), particularly penicillin-binding protein 5 (PBP5) and PBPA. Reduced β-lactam affinity of PBP5 allows cell wall synthesis to continue despite antibiotic exposure (3), while PBPA alters cell wall responses to CPHs (4). Deletion of either protein markedly increases β-lactam susceptibility (3, 4). Conversely, loss of PBPF and PonA, two class-A PBPs, reduces ceftriaxone (CTX) susceptibility while maintaining ampicillin resistance, suggesting a selective role in β-lactam resistance mechanisms (3).

Two-component regulatory systems further contribute to CPH resistance. The CroRS system, well studied in *Enterococcus faecalis*, regulates genes for cell wall integrity and peptidoglycan turnover. Upon CPH exposure, CroR triggers *pbp4* (*pbp5*) overexpression, enhancing cell wall remodeling and promoting resistance (5–7). The StpA/Stk kinase-phosphatase pair (IreP/IreK in *E. faecalis*) also modulates resistance; mutations enhancing Stk activity or disrupting StpA increase CPH resistance, with StpA loss being particularly impactful in *E. faecium* (8). In *E. faecalis*, *ireK* deletion or combined *ireK*/*ireP* loss markedly reduces resistance (9).

To date, *E. faecium* strains with low minimum inhibitory concentrations (MICs) of CPHs have only been obtained through *in vitro* selection, with no reports of naturally occurring isolates. In a recent study, however, we identified several isolates from bacteremic patients belonging to different sequence types (STs), with unexpectedly low CPH MICs (10). Here, we investigate one such clinical isolate as a proof of concept to determine whether genetic changes can modulate CPH susceptibility in a naturally occurring low-MIC background. Using comparative genomic analysis, we show that non-synonymous mutations in *croS*, *nusG*, or *rpoB* restore high-level CPH resistance to the natural isolate with low-MICs, shedding light on previously unrecognized molecular mechanisms that modulate CPH susceptibility in this clinically important pathogen.

## MATERIALS AND METHODS

### Isolation and clinical context of the *E. faecium* Efm5 strain

A low-CPH MIC *E. faecium* clinical strain, designated Efm5, was isolated from an 89-year-old female patient with bacteremia at Parc Taulí Hospital in 2016 (10). The patient had a history of cerebrovascular accident with complete dependence, a permanent urinary catheter, and a doubtful penicillin allergy. She presented with fever and possible cough; urinary and respiratory tract infections were suspected. Empirical treatment with levofloxacin and clindamycin was initiated. After 24 h, blood cultures were positive in two out of four bottles: one grew *Escherichia coli*, and another yielded *E. coli* plus *E. faecium*. The final diagnosis was healthcare-associated bacteremia, most likely of urinary origin. Based on broth microdilution results, treatment was switched to ceftriaxone (the patient had previously tolerated cefuroxime). Although *E. coli* was susceptible to CPHs, no susceptibility testing was performed for *E. faecium*. Since this species is intrinsically resistant to CPHs, the treatment decision can be considered inappropriate. Nevertheless, the infection resolved without further complications.

### Antibiotic susceptibility testing

For research purposes, antibiotic susceptibility tests were conducted using Etest for ampicillin, ceftaroline, CTX, cefotaxime, cefoxitin, vancomycin, and rifampicin. Briefly, a 0.5 McFarland inoculum was prepared using a PhoenixSpec Nephelometer (Becton Dickinson). A sterile cotton swab moistened with the standardized bacterial suspension was then used to inoculate cation-adjusted Mueller–Hinton Agar 2 plates (Millipore), onto which Etest strips (bioMérieux) were applied. Plates were incubated at 37°C, and results were read after 16–20 h of incubation (11). Clinical breakpoints for ampicillin, vancomycin, and rifampicin were interpreted following the Clinical and Laboratory Standards Institute (12). Clinical breakpoints are not available for CPHs.

### Recovery of CPH high-MIC isolates

One hundred microliters of an Efm5 suspension (10^8^ CFU/mL) from an overnight culture grown without antibiotic selection in cation-adjusted Mueller–Hinton Broth 2 (MHB, Millipore) medium was seeded onto Brain Heart Infusion (BHI, Thermo Fisher Scientific) agar plates supplemented with 100 mg/L CTX and incubated at 37°C for 48 h. Colonies growing in such CTX concentration, from now on called CPH high-MIC isolates, were subsequently selected and subcultured onto plates supplemented with 100 mg/L CTX. Frequency of CPH high-MIC variants was determined by dividing the CFUs counted on BHI agar supplemented with 100 mg/L CTX by the total CFU count detected on BHI agar without antibiotics. CTX was selected as a clinically relevant third-generation cephalosporin commonly used in patient care and previously used to select CPH-resistant *E. faecium* derivatives (8).

### Whole-genome sequencing

Whole-genome sequencing (WGS) of Efm5 and the CPH high-MIC variants was performed using Illumina technology, following the pipeline previously described by our group (10). Multilocus sequence typing (MLST) was performed using the MLST software (https://github.com/tseemann/mlst), based on the recently published MLST scheme for *E. faecium* (13). Putative acquired antibiotic resistance genes were predicted with ABRicate (https://github.com/tseemann/abricate) using the in-built NCBI AMRFinderPlus (14). Prediction of the ampicillin resistance phenotype due to *pbp5* mutations was performed with ResFinder, and virulence genes were assessed with Virulence Finder, both from the Center for Genomic Epidemiology (15, 16).

### Comparative genomics

To identify potential transposon insertions or premature stop codons responsible for the CPH low-MIC observed in the Efm5 isolate, a BLASTP analysis was performed using the following protein sequences from the *E. faecium* RefSeq reference genome (SRR24) as the queries: *pbp5* (E6A31_RS06825), *pbpA* (E6A31_RS03475), *croR* (E6A31_RS13520), *croS* (E6A31_RS13525), *stpA* (E6A31_RS12560), *stk* (E6A31_RS12555), and *murAA* (E6A31_RS09060), previously reported as key contributors to intrinsic resistance to CPH (4, 8, 17, 18).

On the other hand, sequencing reads of the CPH high-MIC variants were mapped against the Efm5 genome with Snippy (https://github.com/tseemann/snippy). Synonymous single-nucleotide polymorphisms (SNPs) were discarded for further analysis. Predicted mutations were confirmed using Sanger sequencing (Servei de Genòmica i Espectroscòpia de Biomolècules, Universitat Autònoma de Barcelona).

To gain insights into the global distribution of observed mutations, CroS, NusG, and RpoB homologs were compiled from the 356 complete *E. faecium* assemblies available in the NCBI database. This analysis was conducted through reciprocal BLASTP (19) using the *E. faecium* Efm5 CroS, NusG, and RpoB protein sequences as the queries, a conservative e-value of <1e-20 and query coverage of >75%, similar to that previously described by our group (20). Protein multiple sequence alignments of predicted homologs were performed with CLUSTALW using default parameters (21).

To identify putative regulatory motifs on the *pbp5* operon, FtsW orthologs were identified in complete *E. faecium* genome assemblies available in NCBI, as described above. The upstream regions (from −250 to +2 bp of the predicted translational start site) of identified FtsW orthologs were obtained from the respective complete genome sequences. Redundant upstream sequences (those with nucleotide sequence identity >90%) were removed with USEARCH (22), and the resulting non-redundant panel was used to perform motif discovery. Palindromic motifs were inferred with MEME (23) using a 12–26 bp motif size, the any number of repetitions site distribution model, and otherwise default parameters.

### Growth curves

*E. faecium* growth curves were performed on 96-well microtiter plates with BHI in a Multiskan FC microplate photometer (Thermo Fisher Scientific) at 37°C under constant shaking for 24 h. OD_600_ was recorded every 30 min. Growth rate (μ, h⁻¹) was computed as the slope of the linear regression of ln(OD_600_) versus time during the exponential growth phase. Data are presented as the mean of six replicates.

### *In vitro* time–kill curves

Time–kill curves (TKCs) were conducted in MHB with (10 mg/L) and without (Sigma Aldrich) CTX, using an initial inoculum of 10^6^ CFU/mL. Efm5 and three representative CPH high-MIC variants were incubated at 35°C and 150 rpm. Aliquots of 100 μL were taken at 0, 2, 4, 6, and 24 h for each strain and condition, and 10 μL of each aliquot was plated in duplicate on BHI agar, which was then incubated at 37°C for 24 h to determine bacterial viability, expressed as CFUs per milliliter. Experiments were performed in triplicate following standard procedures, and the average of counts was considered (24).

### Immunoblotting

Whole cell lysates were prepared from exponential cultures of *E. faecium* Efm5, the five CPH high-MIC variants and GE-1 (a *pbp5*-negative mutant), grown on MHB. Samples were heated at 100°C for 10 min, mixed with Laemmli sample buffer, and normalized so that the total protein concentrations were equal. Then, samples were electrophoresed on 10% polyacrylamide gels by standard procedures. Gels were stained with Coomassie blue or transferred electrophoretically to nitrocellulose membranes following the manufacturer’s protocol. Membranes were then probed with rPBP5-S polyclonal rat sera (1:2,000) followed by HRP-conjugated goat anti-rat IgG antibodies (1:2,000) (RAb-035 Neo Biotech) and developed using Luminata Forte Western HRP substrate (Millipore) following the manufacturer’s instructions. In addition to the band corresponding to PBP5 (~70 kDa), a non-specific lower-molecular-mass band was detected and used as an internal loading control. Western blot analyses were performed in duplicate.

### Transcriptional and transcriptomic analysis

#### RNA extraction

Exponential cultures of Efm5 and all the CPH high-MIC variants were grown on MHB without antibiotics at 37°C and 150 rpm. Total RNA was purified using the RNeasy Mini Kit (Qiagen) according to previously published methods (7). At the end, RNA was treated with TURBO DNase (Thermo Fisher Scientific) according to the manufacturer’s recommended protocol. Concentration and purity of the extracted RNA were assessed using an RNA Nano Chip (Agilent Technologies).

#### RT-qPCR analysis

RT-qPCR was employed to assess gene expression. cDNA synthesis was carried out using the iScript cDNA Synthesis (Bio-Rad) kit. qPCR was performed with iTaq polymerase (Bio-Rad) and SYBR green in a CFX96 PCR instrument (Bio-Rad). Relative gene expression was calculated using the Pfaffl method (25). Differential gene expression was judged based on the common arbitrary twofold cutoff using *adk*, *gyrB*, and *ddl* as housekeeping genes. Data presented in the article correspond to the analysis of RNAs isolated from three independent biological repeats. Primers used for qPCR were designed using Primer3 software and are listed in Table S1.

#### RNA sequencing

The RNA library was prepared using the Illumina Stranded Total RNA Prep Ligation with Ribo-Zero Plus kit and 10 bp unique dual indices. The sequencing was carried out on a NovaSeq X Plus (SeqCenter, USA), generating paired-end 150 bp reads. At least four biological replicates were analyzed per strain.

#### Differential expression analysis

The quality of raw sequencing data were assessed using FastQC (https://github.com/s-andrews/FastQC), followed by trimming using Trimmomatic (https://github.com/usadellab/Trimmomatic). Residual rRNA reads were then eliminated using the SortMeRNA software (26). Filtered reads were then pseudoaligned against the *E. faecium* Efm5 transcriptome for transcript-level quantification using Salmon (27). Differential expression gene (DEG) analysis was finally performed with the DESeq2 Bioconductor R package (28). Genes showing −1.5 ≤ log_2_ fold change ≥1.5 and adjusted *P* value of <0.05 were considered as DEGs.

### Electron microscopy

For transmission electron microscopy (TEM) examination, exponential-phase cultures of *E. faecium* Efm5 and three representative CPH high-MIC variants, grown in BHI broth without antibiotics for 2.5 h at 37°C and 150 rpm, were fixed in a solution containing 2.5% glutaraldehyde in 0.1 M phosphate buffer for 2 h at 4°C. Then, they were post-fixed in a solution containing 1% osmium tetroxide with 0.8% potassium ferrocyanide for an additional 2 h. Subsequently, samples underwent dehydration using a series of increasing ethanol concentrations (50%, 70%, 90%, 96%, and 100%). Next, the cell pellets were embedded in EPON Epoxy Resin (EMS, Hatfield, PA, USA) and polymerized at 60°C for 48 h. Thin sections with a thickness of 70 nm were obtained using a Reichert-Jung Ultracut E ultramicrotome. These sections were then strained with 2% uranyl acetate and Reynold’s solution (0.2% sodium citrate and 0.2% lead nitrate), followed by examination under a Hitachi H-7000 transmission electron microscope operating at a voltage of 75 kV. All chemicals and reagents were procured from Sigma Chemical Co. (St. Louis, MO, USA), unless otherwise specified. At least 20 micrographs of each strain were taken and analyzed. Given the particular features observed in the *rpoB*-R5 variant, three independent preparations were analyzed.

TEM images were analyzed using Fiji (29). Electron-lucent regions were quantified by applying a visually optimized threshold to each cell to generate binary masks, with electron-lucent areas identified as white pixels and expressed as a percentage of total cell area. Bacterial cell wall thickness was measured by manually drawing a line perpendicular to the cell wall. Analyses were performed on representative cells of comparable size and morphology using images acquired at identical magnifications: 20× for electron-lucent area quantification (≥50 cells per strain) and 40× for cell wall thickness measurements (15–20 cells per strain). Statistical significance was assessed using the Mann–Whitney *U* test.

## RESULTS

### Low cephalosporin MICs observed in *Enterococcus faecium* Efm5 isolate

WGS analysis identified the *E. faecium* Efm5 strain as ST1195 according to PubMLST for *E. faecium*. It was susceptible to ampicillin (0.25 mg/L) and vancomycin (MIC = 3 mg/L) and resistant to rifampicin (MIC = 8 mg/L). It also exhibited low MICs to ceftaroline (0.19 mg/L), cefotaxime (1 mg/L), and CTX (3 mg/L) (Table 1).

**TABLE 1:** Minimum inhibitory concentrations (MICs, mg/mL) of several antibiotics of the *Enterococcus faecium* Efm5 strain and in representative high-CPH MIC variants (*croS-*R1, *nusG*-R3, and *rpoB*-R5)

Strains	Ampicillin	Ceftaroline	Ceftriaxone	Cefotaxime	Cefoxitin	Rifampicin	Vancomycin
Efm5	0.25	0.19	3	1	48	8	3
*croS*-R1	1	0.5	>256	>32	128	N/A^*a*^	4
*nusG*-R3	0.38	0.19	>256	>32	64	N/A	2
*rpoB*-R5	1.5	0.5	>256	>32	64	>32	2

^*a*^ N/A, not applicable.

Several acquired resistance genes were predicted, including those conferring resistance to chloramphenicol (*catA8*), macrolides [*msr(C)*], and tetracyclines [*tet(L)* and *tet(M)*]. Notably, genes associated with CPH resistance (*pbp5*, *pbpA*, *croRS*, *stpA-stk*, *murAA*) were identified without any transposon insertions or nonsense mutations that could lead to premature protein termination. However, our *in silico* analyses revealed a nonsense mutation in the *psr* gene. Mutations in PBP5 predicted to be associated with ampicillin resistance by ResFinder included V24A, S27G, R34Q, G66E, E100Q, K144Q, T172A, L177I, A216S, T324A, N496K, A499I, and E525D. VirulenceFinder analysis revealed the presence of multiple virulence factor-encoding genes, such as *acm*, *bepA*, *ccpA*, *empABC*, *fms13*, *fms14*, *fms15*, *fms17*, *fnm*, *sagA*, and *scm*.

### Single-point mutations drive cephalosporin high-MIC variants of *Enterococcus faecium* Efm5

Five CPH high-MIC colony variants (R1–R5) of Efm5 were detected in BHI agar plates supplemented with CTX (100 mg/L), yielding an isolation frequency of 5 × 10⁻⁷. Etest results confirmed that they exhibited elevated MICs to CTX (>256 mg/L), cefotaxime (MIC >32 mg/L), and cefoxitin (>64 mg/L) compared to the parental Efm5 strain (Table 1). However, all variants remained susceptible to ampicillin and vancomycin, with low MICs of ceftaroline. Notably, the R5 variant also exhibited a fourfold increase in the MIC of rifampicin (Table 1).

**Fig 1:**
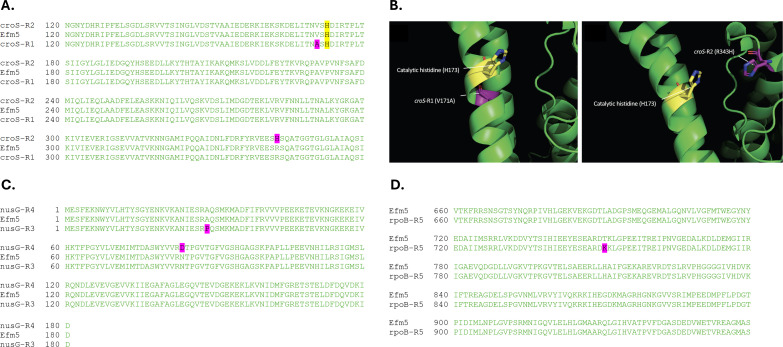
(**A**) Segment of the CroS multiple sequence alignment encoded in Efm5, *croS*-R1, and *croS*-R2 variants. The catalytic histidine (H173) is highlighted in yellow. (**B**) Visual representation of the CroS protein 3D structure for the *croS*-R1 (left) and *croS*-R2 (right) variants, generated by AlphaFold (Q3XYJ6) and visualized with PyMOL. (**C**) NusG multiple sequence alignment encoded in Efm5, *nusG*-R3, and *nusG*-R4 variants. (**D**) Segment of the RpoB multiple sequence alignment encoded Efm5 and the *rpoB*-R5 variant. In all panels, predicted mutations are labeled in purple.

Comparative genomics revealed that each CPH high-MIC variant harbored one SNP compared to the parental Efm5 strain. Specifically, the R1 and R2 variants contained point mutations in the *croS* gene, resulting in V171A (*croS-*R1 variant) and R343H (*croS-*R2 variant) substitutions in the CroS protein (Fig. 1A). Structural analysis using AlphaFold predictions indicated that both V171 and R343 are located near the phosphoryl-accepting residue H173 of CroS, which is critical for the activity of this regulatory protein (Fig. 1B) (5).

The R3 and R4 variants presented mutations in the *nusG* gene, leading to A29P (*nusG-*R3 variant) and N84D (*nusG-*R4 variant) substitutions in the NusG protein (Fig. 1C). NusG is a conserved intrinsic transcription termination factor essential for transcriptional regulation in bacteria (30). Finally, the rifampicin-resistant R5 variant exhibited a T798K substitution in the RpoB protein (Fig. 1D), which corresponds to the β-subunit of RNA polymerase (*rpoB*-R5 variant).

The search for homologs of such variants of CroS, NusG, and RpoB in all complete NCBI *E. faecium* genome assemblies revealed that over 99.0% of genomes encoded *croS*, *nusG*, and *rpoB* genes. Identical NusG, RpoB, and CroS protein sequences to those found in the Efm5 strain were identified in 99.4%, 76.7%, and 85.9% of the *E. faecium* genomes analyzed, respectively. However, NusG, RpoB, and CroS sequence variants associated with high MIC values of CPHs in our isolates were not detected in any of the analyzed genomes in the NCBI database (Table S2).

### Differential growth of *Enterococcus faecium* Efm5 and high-MIC variants

*E. faecium* Efm5 and the high-MIC variant isolates exhibited similar endpoints and growth dynamics (Fig. S1), with growth rates (μ) ranging from 0.67 to 0.77 h⁻¹. The exponential phase spanned the 3–5 h timepoints.

**Fig 2:**
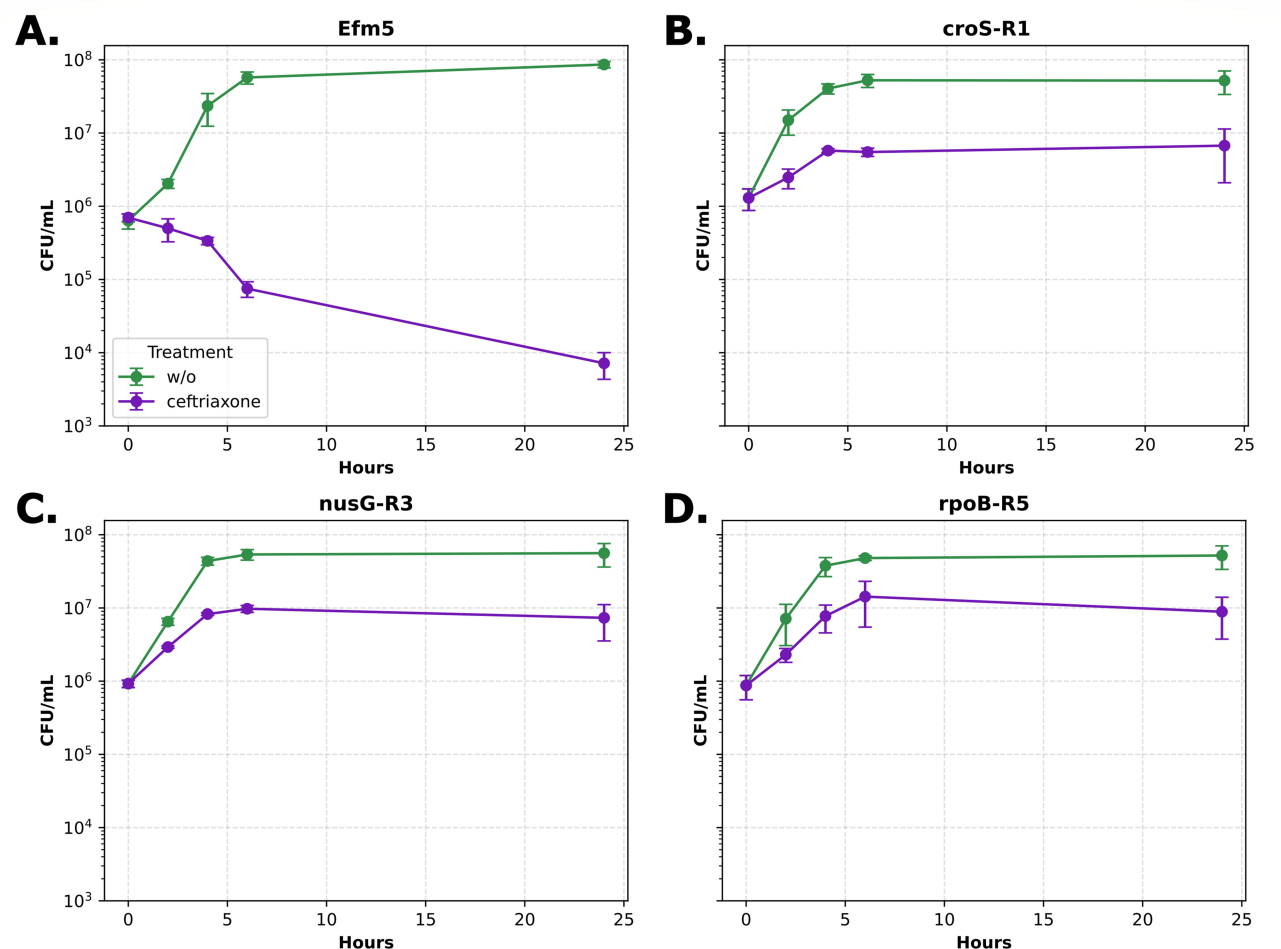
Growth curves of the (**A**) CPH low-MIC Efm5 and the CPH high-MIC variants, (**B**) *croS*-R1, (**C**) *nusG*-R3, and (**D**) *rpoB*-R5, both in the absence and presence of ceftriaxone (10 mg/L). Standard deviations are represented by error bars.

Time–kill curves were conducted in the presence of 10 mg/L of CTX. The results confirmed the predicted inhibitory effect of CTX on Efm5 (Fig. 2A). In contrast, the *croS*-R1 (Fig. 2B), *nusG*-R3 (Fig. 2C), and *rpoB*-R5 (Fig. 2D) variants were all able to grow in the presence of CTX.

### Increased PBP5 expression in CPH high-MIC variants

**Fig 3:**
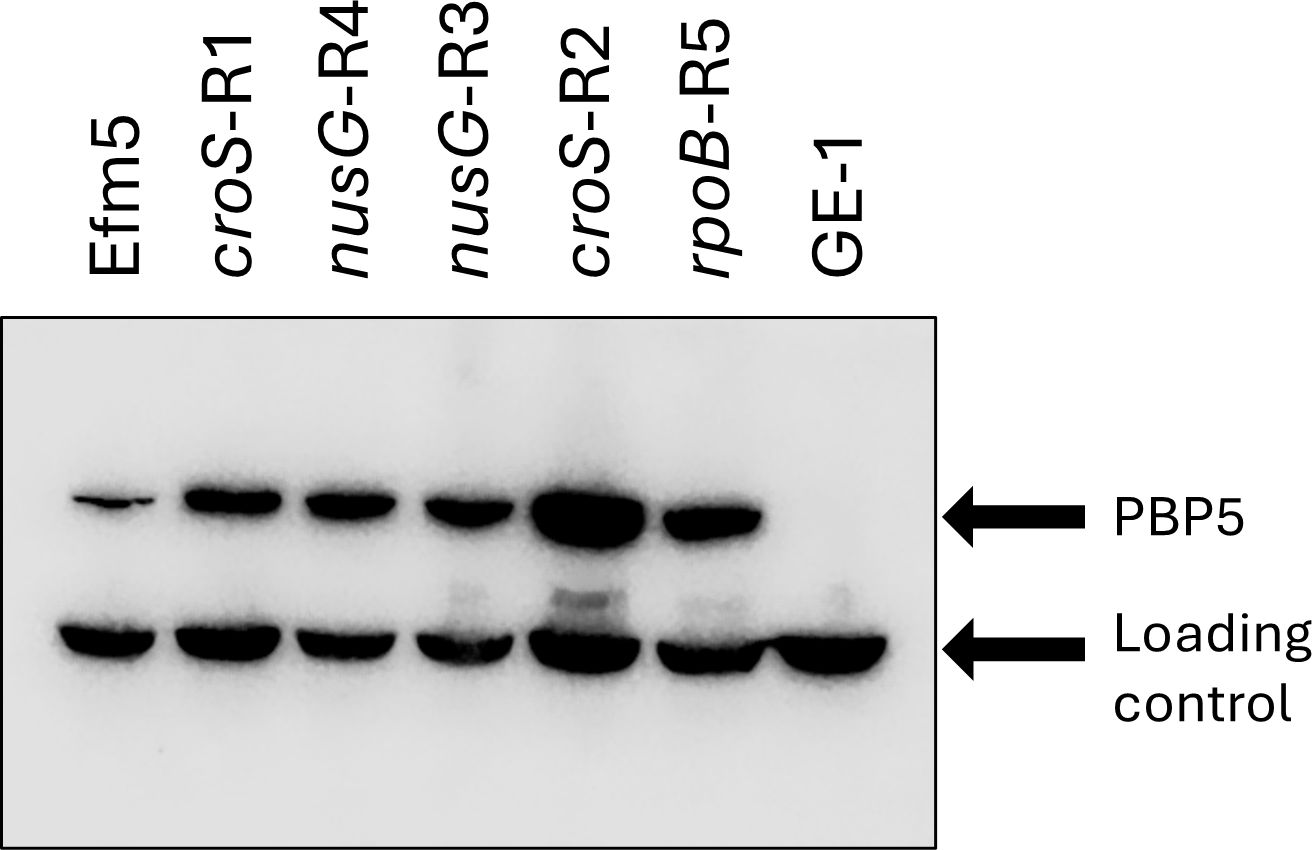
Western blotting of *E. faecium* Efm5, five CPH high-MIC variants, and GE-1 (a *pbp5*-negative mutant) using rPBP5-S polyclonal rat sera. In addition to the band corresponding to PBP5 (~70 kDa), a non-specific lower-molecular-mass band was detected and used as an internal loading control.

As PBP5 is the primary determinant of CPH resistance in *E. faecium* (3), its expression was assessed via Western blotting using an anti-PBP5 monoclonal antibody (31). Our results revealed increased levels of PBP5 expression in all the CPH high-MIC variants compared to the Efm5 strain (Fig. 3; Fig. S2). No PBP5 was detected in *E. faecium* GE-1, a *pbp5*-negative strain (32).

**Fig 4:**
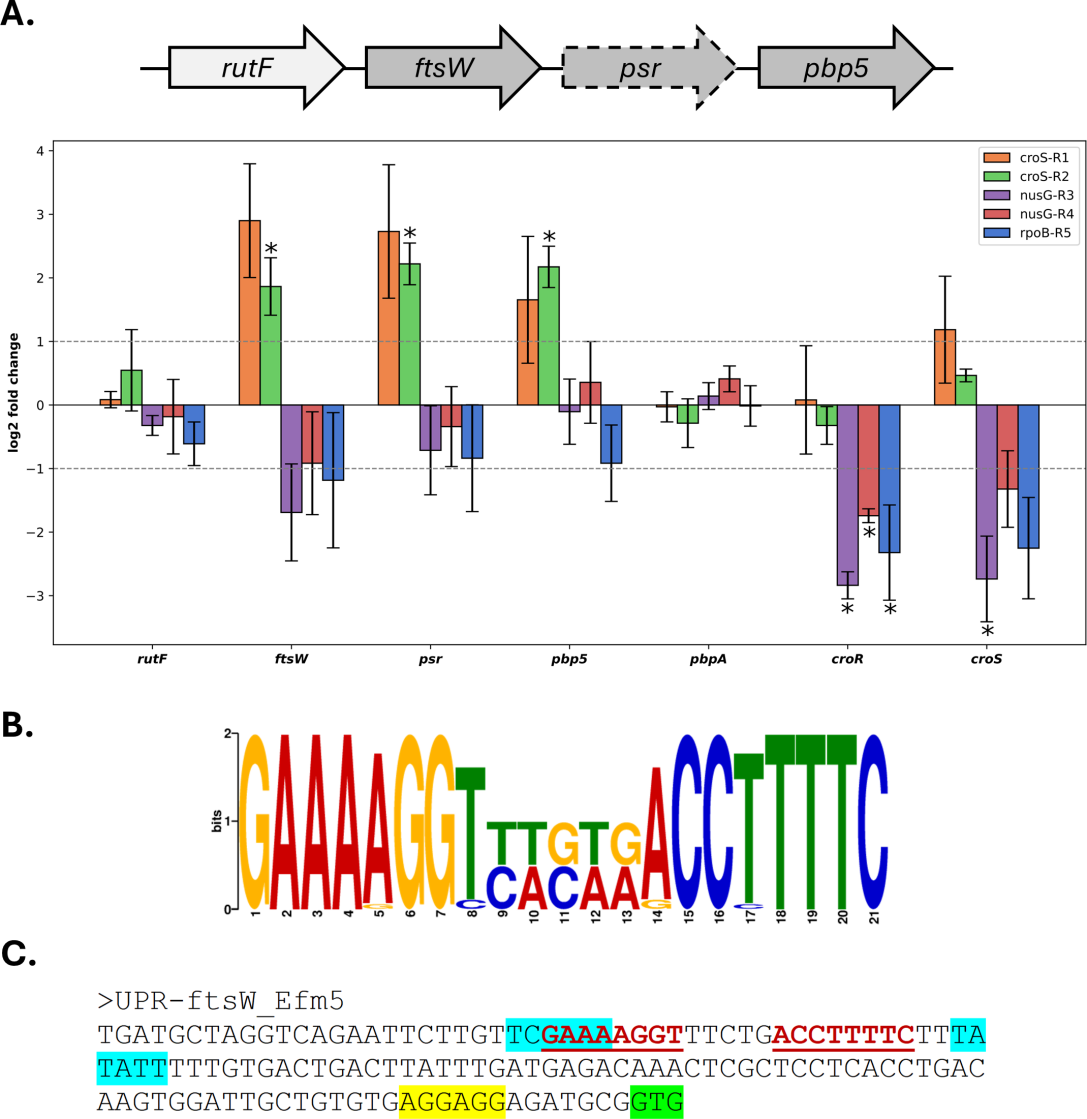
(**A**) Transcriptional analysis of the *pbp5* operon in CPH high-MIC variants compared to the *E. faecium* Efm5 isolate. Fold-change values (log_2_) for the three genes of the *pbp5* operon (*ftsW, psr*, and *pbp5*), *rutF* (located immediately upstream of the operon), and the regulatory genes *croR* and *croS* are shown. Dashed lines indicate biologically relevant fold-change thresholds. Asterisks highlight genes with statistically significant expression differences relative to the Efm5 strain. (**B**) Sequence logo of the GAAAAGGT-N5-ACCTTTTC motif identified by MEME upstream of genes encoding FtsW proteins in *E. faecium*. (**C**) Schematic representation of the upstream region of the *ftsW* gene in Efm5, illustrating putative regulatory elements. The −35 and −10 promoter sequences are highlighted in light blue, the ribosome binding site in yellow, and the translation start codon in light green. The DNA motif identified by MEME is highlighted in red and underlined.

The increased PBP5 expression observed by Western blotting in the CPH high-MIC variants prompted us to analyze transcriptional changes within the *pbp5* operon using RT-qPCR. In the *croS*-R1 and *croS*-R2 CPH high-MIC variants, this analysis revealed a significant upregulation of the *ftsW*, *psr*, and *pbp5* genes (Fig. 4A). Transcription of *rutF*, located immediately upstream of the operon, remained unchanged. Additionally, no significant changes were observed in the transcript levels of *pbpA* or *croR*, although *croS* expression was slightly activated in the *croS-*R1 variant. In contrast, no transcriptional changes in the *pbp5* operon were detected in the *nusG-*R3, *nusG-*R4, and *rpoB-*R5 variants, although these variants exhibited downregulation of *croS* and *croR*. The *nusG-*R4 and *rpoB-*R5 variants also showed slightly repressed expression of *ftsW*.

These findings prompted us to examine the upstream region of *ftsW*, the first gene of the *pbp5* operon, for potential regulatory sequences. Analysis of the upstream regions of all genes encoding FtsW homologs across complete *E. faecium* genome assemblies available in NCBI identified a significant motif with palindromic structure (GAAAAGGT-N5-ACCTTTTC) (Fig. 4B; Table S3). This motif was conserved in the *ftsW* promoters of Efm5 and all CPH high-MIC variants (Fig. 4C). In these promoters, the predicted −35 (TCGAAA) and −10 (TATATT) regions were located 101 and 76 nucleotides upstream of the *ftsW* translational start codon, respectively (Fig. 4C). As it is typical for canonical promoters controlled by the vegetative sigma factor, the −35 and −10 regions were spaced 19 bp apart. The identified motif was situated near the −35 and −10 elements, suggesting a potential role in modulating RNA polymerase binding and, consequently, transcription of the *pbp5* operon.

### Global functional and metabolic reprogramming in CPH high-MIC variants

**Fig 5:**
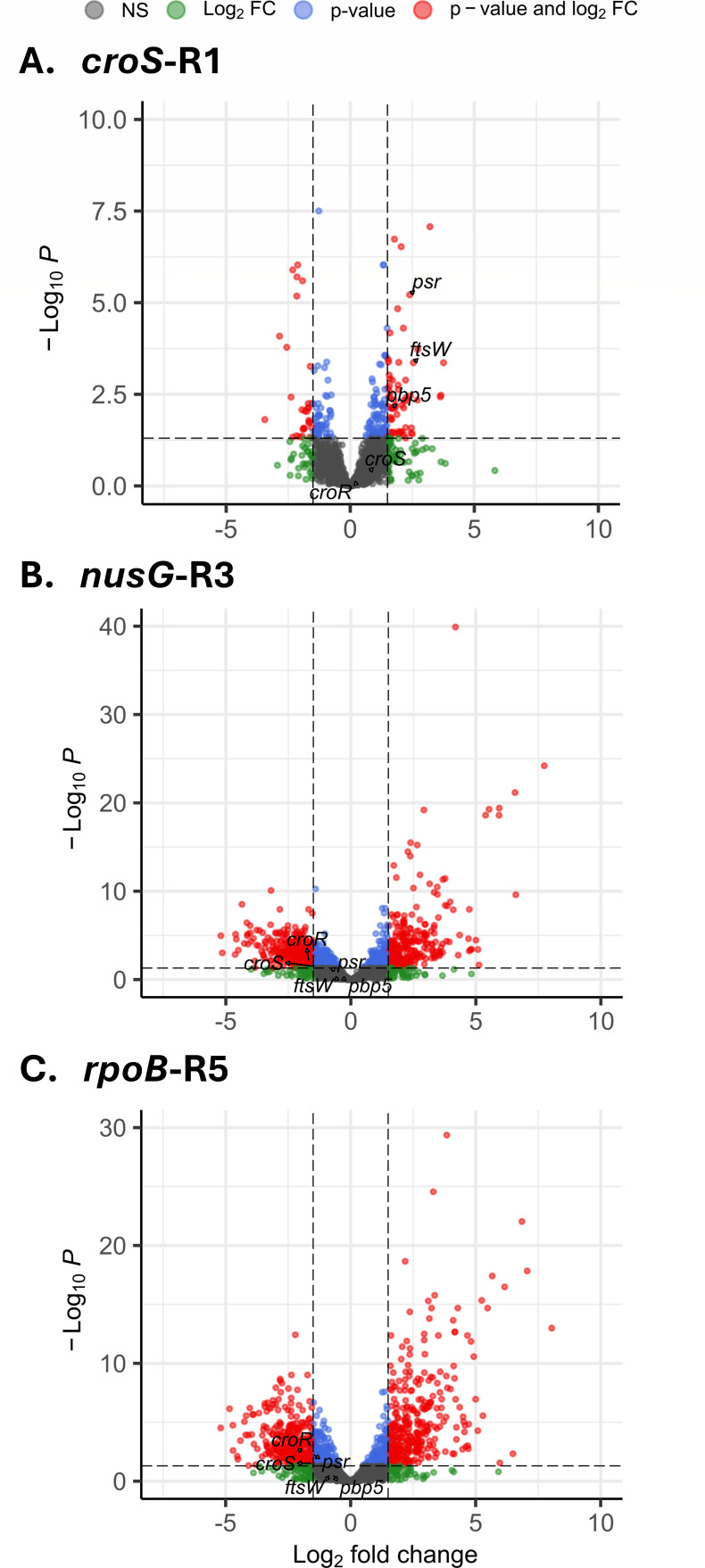
Volcano plots showing differentially expressed genes when comparing *croS*-R1 (**A**), *nusG*-R3 (**B**), and *rpoB*-R5 (**C**) variants against Efm5. Dots corresponding to *ftsW*, *psr*, *pbp5*, *croR*, and *croS* genes are highlighted.

Genome-wide transcriptional changes in the high-MIC variants compared to the Efm5 strain were assessed by RNA-seq. The *croS*-R1 transcriptome revealed 90 differentially expressed genes (DEGs), including 61 upregulated and 29 downregulated genes (Fig. 5A; Table S4). The results showed coordinated activation of genes mainly involved in cell envelope biogenesis, carbohydrate utilization, translation, and nutrient transport. Genes encoding peptidoglycan synthesis and cell wall remodeling enzymes (*ftsW*, *pbp5*, *pbp2A*, *dltC*, and *uppP*) were also upregulated, consistent with RT-qPCR data (Fig. 4A). The role of CroRS in regulating the expression of low-affinity PBPs in *Enterococcus* has been previously documented (6, 7).

RNA-seq analysis of the *nusG*-R3 (Fig. 5B) and *rpoB*-R5 (Fig. 5C) confirmed constitutive expression of *pbp5* and decreased levels of *croS* and *croR* transcription observed by RT-qPCR. In both cases, transcriptional changes were extensive and indicative of global functional and metabolic reprogramming. Transcriptional profiling of the *nusG*-R3 variant identified 612 DEGs, including 290 upregulated and 322 downregulated genes (Fig. 5B; Table S5). The transcriptional response was dominated by strong activation of translation-related genes, including ribosomal protein genes (*rpl* and *rps* families) and numerous aminoacyl-tRNA synthetases (*alaS*, *ileS*, *leuS*, *tyrS*, *argS*, *aspS*, *valS*, and *pheS/T*), indicating a major increase in protein synthesis capacity. Conversely, several peptidoglycan and membrane biosynthesis genes (*pbp1B*, *pbp2A*, *pbp2B*, *dacB*, *murE/E2*, *mltG*, *glmU*, and *dapA/B/F/H*) were repressed, along with regulators of antimicrobial resistance and virulence (*mprF*, *phoP*, and *ciaR*). Additional repression affected central metabolism and cofactor biosynthesis (*gap*, *gpsA*, *gdh*, *mvaS/D*, *coaBC*, *guaB*, and *cobQ*) and nutrient transporters (*ugpA7*, *azlC*, *fruK*, *licC*, and *srlB*).

The *rpoB*-R5 variant exhibited 666 DEGs, with 336 genes upregulated and 330 downregulated (Fig. 5C; Table S6). Overall, its transcriptional profile was similar to that of the *nusG*-R3 variant and was dominated by the activation of protein synthesis and cell remodeling pathways. Increased expression was observed for ribosomal proteins (*rpl* and *rps* families), translation initiation and elongation factors (*infA/B*, *fusA*, *tsf*, and *metG*), and tRNA synthetases (*alaS*, *leuS*, and *pheS*). Central metabolic and biosynthetic genes, including those involved in fatty acid biosynthesis (*fabD/F/G/Z *and *accA-accD*), amino acid biosynthesis (*metW*, *asnB*, *aroB/E*, and *glnA*), nucleotide biosynthesis (*guaC*, *pyrG*, *purB*, and *apt*), and energy metabolism (*ackA*, *sfcA*, *ndh*, and *glpD*) were also upregulated. Genes associated with DNA replication and repair (*polC*, *topA*, *comFA*, *dinG*, *mutS1*, *radC*, and *ssb*), stress response and redox management (*hslO*, *dpsB*, *zur*, *sigH*, and *cspA*), and cell envelope remodeling (*murF*) were similarly activated. In contrast, regulatory and signaling proteins (*phoP*, *rpoN*, *ctsR*, and *spxA*), oxidative stress and universal stress proteins (*ahpC/F*, *msrA/B*, and *ciaR*), cell envelope modification enzymes (*pbp1B*, *pbp2A*, *gpsB*, *dltA*, and *mprF*), redox and metal homeostasis proteins (*copY*, *rex*, *fer*, and *corA*), and amino acid or cofactor biosynthesis genes (c*ysA/K*, *dapA/B/F/H*, *lysA/C*, *thiI*, *nadE*, and *queH*) were downregulated.

### Distinct ultrastructural features in *E. faecium* Efm5 and cephalosporin high-MIC variants

**Fig 6:**
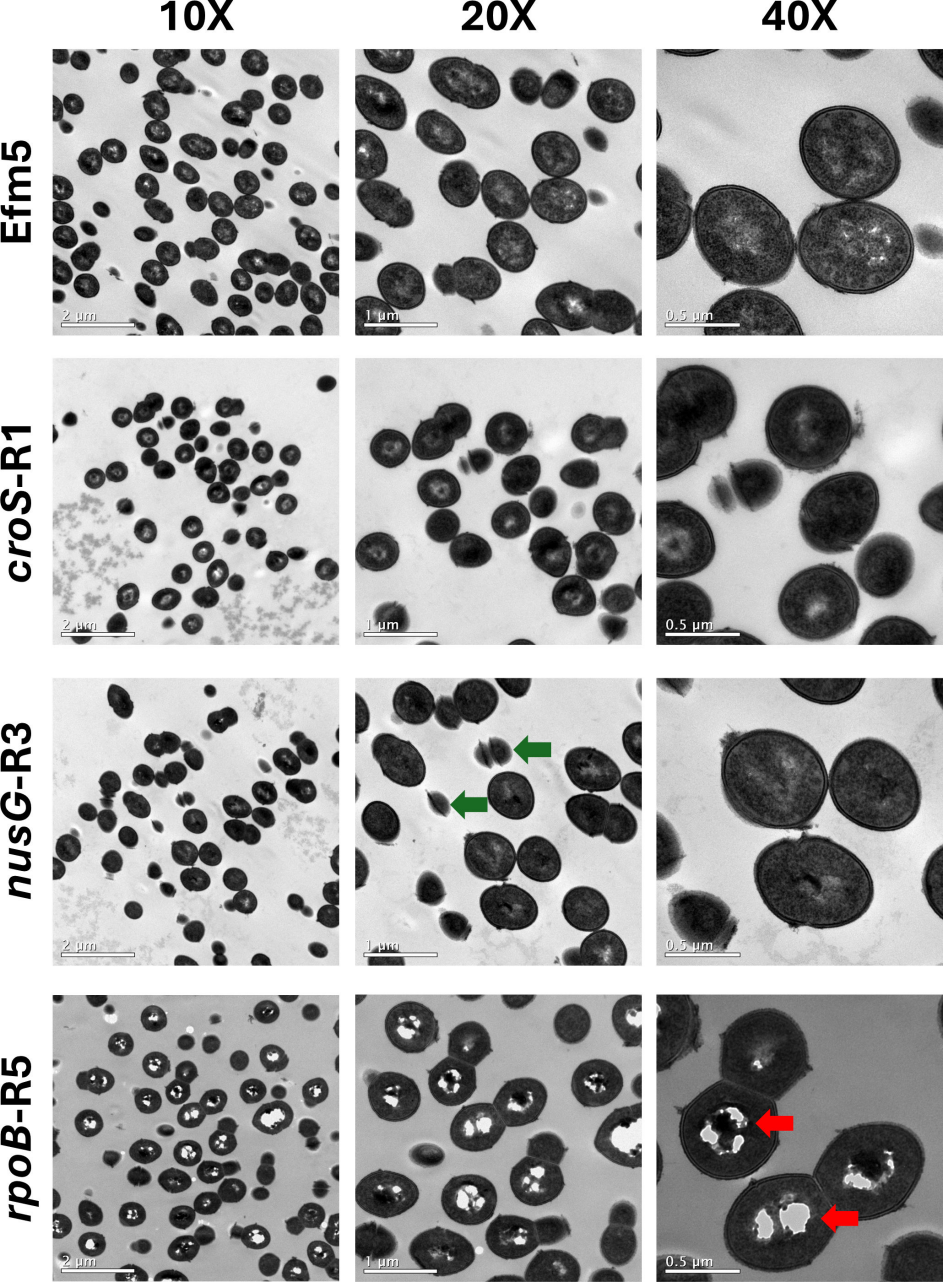
Transmission electron microscopy micrographs of Efm5 and CPH high-MIC variants, captured from the same field at 10×, 20×, and 40× magnification. Red arrows highlight representative electron-lucent regions, and green arrows indicate selected small round bodies with an electron-dense cross-like structure.

TEM was performed to investigate the ultrastructural features of the strains (Fig. 6; Fig. S3). Quantitative analysis did not reveal significant differences in cell wall thickness between Efm5 and the high-CPH-MIC variants (Fig. S4A). Notably, the *rpoB*-R5 variant displayed prominent electron-lucent cytoplasmic regions, visible as clear intracellular zones. These electron-lucent regions were consistently observed across independent preparations of the *rpoB*-R5 strain. Quantitative analyses showed that *rpoB*-R5 harbored a higher percentage of electron-lucent cytoplasmic area (7.5% ± 6.5 SD) compared with Efm5 (1.7% ± 1.5 SD), *croS*-R1 (2.1% ± 1.9 SD), and *nusG*-R3 (0.9% ± 1.3 SD) (Fig. S4B). In addition, the three CPH high-MIC variants exhibited small round bodies with an electron-dense cross-like structure. These structures were detected in 3.9% of Efm5 cells, compared with 7.1% in *rpoB*-R5, 8.5% in *croS*-R1, and 9.5% in *nusG*-R3.

## DISCUSSION

CPH resistance in *E. faecium* is traditionally considered an intrinsic and stable trait, yet the genetic and regulatory processes that shape this phenotype remain incompletely understood. The identification of a clinical *E. faecium* isolate (Efm5) with unusually low CPH MICs provides a unique opportunity to examine how this resistance state can be modulated in a natural genetic background. Our findings indicate that CPH susceptibility in *E. faecium* is not fixed but can shift rapidly through limited genetic changes that impact global regulatory and cell envelope-associated pathways. This plasticity highlights the central role of regulatory networks, rather than structural resistance determinants alone, in controlling β-lactam responses in this species.

Despite ResFinder predicting ampicillin resistance-associated mutations, Efm5 was phenotypically susceptible, indicating that some *pbp5* mutations do not reliably predict resistance. Evidence suggests that only specific substitutions (E629V, 466′S/D, and M485A/T) are key determinants of ampicillin non-susceptibility (33, 34). The absence of such mutations in Efm5 is therefore consistent with its susceptible phenotype. The antimicrobial susceptibility profile of Efm5 was otherwise concordant with its resistome, as erythromycin resistance correlated with the presence of *msr(C*). Although additional resistance genes [*catA8* and *tet(L)*/*tet(M)*] were identified, corresponding phenotypic testing was not performed. Efm5 also harbored a combination of virulence determinants associated with both clinical and non-clinical *E. faecium* lineages, suggesting a mixed genetic background and highlighting the potential for gene exchange across ecological niches (16).

Selection of high-level CPH-resistant variants from *E. faecium* Efm5 under antibiotic pressure demonstrated that marked increases in MIC can arise through discrete genetic events. Notably, these increases were not accompanied by reduced susceptibility to ampicillin or ceftaroline, indicating the involvement of resistance mechanisms beyond canonical alterations in PBP5. The recurrent identification of non-synonymous mutations in *croS*, *nusG*, and *rpoB* among independently selected resistant variants supports a multifactorial basis for CPH resistance in *E. faecium*. Given the intrinsic resistance of enterococci to CPHs, the absence of these mutations from publicly available *E. faecium* genomes associated with high CPH MICs suggests that they may represent compensatory changes contributing to enhanced CPH resistance in the Efm5 background. Comparable growth rates between resistant variants and the parental strain indicate no detectable fitness cost under the *in vitro* conditions tested; however, fitness effects may emerge *in vivo* under additional selective pressures.

In particular, the *croS* mutations V171A (*croS*-R1) and R343H (*croS*-R2) were located near the key phosphoryl-accepting residue H173, suggesting an impact on signal transduction within the CroRS two-component system, a known regulator of genes involved in lipid II biosynthesis, including the *pbp5* operon (6, 7). Consistent with a regulatory disruption, both CroS variants showed increased transcription of *pbp5*, *ftsW*, and *psr*, along with elevated PBP5 protein levels, even in the absence of antibiotic pressure. These results were corroborated by RNA-seq analysis, which revealed a transcriptional landscape indicative of a metabolic shift toward cell envelope remodeling and enhanced nutrient acquisition. This profile is consistent with adaptation to envelope stress and would suggest that the V171A change identified in the *croS*-R1 isolate may result in a constitutively active CroS. The involvement of the CroRS system in CPH resistance has been previously reported in *E. faecalis* (5). Of note, recent studies have shown that the CroS extracellular domain is essential for proper signaling, yet it is not involved in ligand binding or direct sensing (35).

Unlike the CroS variants, the *nusG* and *rpoB* mutations appear to drive CPH resistance through distinct, possibly post-transcriptional mechanisms. These variants do not activate transcription of the *pbp5* operon and show a slight downregulation of *croS* and *croR*, along with minor repression of *ftsW*, suggesting an indirect regulatory effect. Nevertheless, they exhibit increased levels of PBP5, indicating an uncoupling between transcript abundance and protein expression. Notably, the *fstW* upstream region harbors putative regulatory sequences overlapping the ribosome binding site, which could mediate a post-transcriptional regulation of the *pbp5* operon (Fig. S5). This pattern suggests that broader regulatory processes beyond the canonical *pbp5*-driven model may be involved in modulating resistance. In keeping with the role of NusG and RpoB as global regulators, RNA-seq analysis revealed extensive reprogramming of cellular processes involving a large number of DEGs, consistent with observations reported for *rpoB* and *nusG* mutants in other bacteria (30, 36). The transcriptional profiles of the *nusG*-R3 and *rpoB*-R5 variants reveal a convergent physiological response marked by strong activation of translational machinery and cell remodeling. This context could provide a permissive environment for PBP5 synthesis and accumulation, contributing to the high-MIC phenotype observed in such strains. Conversely, other genes involved in cell wall and membrane biogenesis, stress responses, virulence regulation, and oxidative stress defenses were broadly downregulated, suggesting a trade-off between growth and stress resilience. Overall, both variants appear to reprogram global transcription to favor translational efficiency and metabolic activity, likely reflecting compensatory adaptation.

Previous studies have shown that *rpoB* mutations (H486Y, H486D, and Q473K) can alter CPH susceptibility in *E. faecium* and *E. faecalis*, although the underlying molecular mechanisms have not yet been fully elucidated (37). Our findings add to this evidence and reinforce the notion that RpoB mutations contribute to antibiotic resistance beyond their well-known association with rifampicin, as also reflected in the observed increase in rifampicin MIC. Furthermore, recent studies have linked RpoB alterations to daptomycin resistance (38), underscoring the pleiotropic effects of this global transcriptional regulator.

The involvement of NusG is particularly intriguing, as this gene encodes a conserved transcription termination factor not previously implicated in antibiotic resistance. Its potential association with altered PBP5 expression suggests a novel link between transcriptional regulation and β-lactam susceptibility, warranting further investigation. The complexity of the *pbp5* promoter region, containing both canonical motifs and additional regulatory elements near the ribosome binding site, is consistent with the existence of multilayered control mechanisms potentially sensitive to global regulatory perturbations.

Structural and morphological defects observed in all variants by TEM, most notably in the *nusG*-R3 and *rpoB*-R5 strains, which exhibited electron-dense bodies, may reflect underlying disruptions in cell division or peptidoglycan remodeling. Such perturbations are consistent with stress-induced morphogenetic responses documented in staphylococci (39), implying that resistance-associated mutations could exert pleiotropic effects impacting cell envelope integrity. The presence of electron-lucent cytoplasmic regions specifically in the *rpoB*-R5 variant further suggests metabolic dysregulation or altered transcriptional activity, reinforcing the concept that these mutations induce complex physiological shifts beyond mere target modification.

To date, our group has identified four *E. faecium* isolates with low MICs to CPH (Efm1, Efm5, Efm10, and Efm54), all obtained from patients with bacteremia (10). Recent evidence has shown that clade A1 *E. faecium* isolates encode a shorter *psr* isoform (757 bp), while clade B isolates (now *Enterococcus lactis*) carry a longer version (885 bp), which has been associated with lower *pbp5* expression and reduced ampicillin MICs (31). Notably, all of the low-MIC isolates from our collection (Efm1, Efm5, Efm10, and Efm54) carried the clade B isoform, which is associated with low PBP5 expression. As described earlier, we found a premature stop codon in *psr* in the Efm5 isolate, raising the possibility that disruption of this gene may also contribute to CPH susceptibility. Specifically, the Psr protein encoded by the Efm5 strain is predicted to be 127 amino acids in length, compared with the 294-amino acid clade B wild-type isoform of *E. faecium* COM15 (31). Nonsense mutations in *psr* were also found in Efm1, which shares the low-MIC phenotype, and in Efm57, which instead displayed the classical intrinsic resistance to these antibiotics (20). However, the presence of the wild-type *psr* gene in the other low-MIC isolates (Efm10 and Efm54) further indicates that *psr* disruption alone is insufficient to explain the observed phenotype. Although a potential contribution of *psr* variants to CPH susceptibility cannot be ruled out, our findings suggest that additional genetic determinants are likely involved. Further studies are needed to elucidate the genetic basis of the low CPH MIC phenotype in *E. faecium*.

In conclusion, we identified previously uncharacterized mutations in *croS*, *nusG*, and *rpoB* that demonstrate how CPH resistance in *E. faecium* can reemerge via multiple evolutionary paths, including modifications in signal transduction systems and global transcriptional regulators. Moreover, we demonstrate the existence of naturally occurring *E. faecium* with low MICs of CPH, which would probably be considered as susceptible if breakpoints were available. The presence of these low-MIC isolates is interesting, and the genetic causes require further attention. The emergence of more low-MIC *E. faecium* strains, such as Efm5, could challenge prevailing assumptions about the intrinsic nature of CPH resistance in this species and offer new perspectives on its adaptive responses to β-lactams in clinical settings. Our findings carry important implications for surveillance and antibiotic stewardship, especially in clinical contexts where CPHs are employed as part of combination therapy against multidrug-resistant enterococcal infections. Overall, our results advance current understanding of enterococcal adaptation to β-lactams and highlight the need to consider atypical resistance phenotypes in diagnostic and therapeutic strategies.

## Data Availability

Whole-genome sequencing and RNA-seq data generated in this study have been deposited in the NCBI database with accession number PRJNA1290041.

## SUPPLEMENTAL MATERIAL

Fig. S1 aac.01948-25-s0001.tiff Supplemental figure 1.

Fig. S2 aac.01948-25-s0002.tiff Supplemental figure 2.

Fig. S3 aac.01948-25-s0003.tiff Supplemental figure 3.

Fig. S4 aac.01948-25-s0004.tiff Supplemental figure 4.

Fig. S5 aac.01948-25-s0005.tiff Supplemental figure 5.

Supplemental tables aac.01948-25-s0006.xlsx Tables S1 to S6.

ASM does not own the copyrights to Supplemental Material that may be linked to, or accessed through, an article. The authors have granted ASM a non-exclusive, world-wide license to publish the Supplemental Material files. Please contact the corresponding author directly for reuse.
