# Supplementary figures and images for: Single point mutations in global regulatory genes restore cephalosporin resistance in a low-MIC Enterococcus faecium natural isolate
Source: Antimicrob Agents Chemother. 2026 Mar 23;70(5):e01948-25. doi: 10.1128/aac.01948-25 (PMC13148053; doi:10.1128/aac.01948-25)

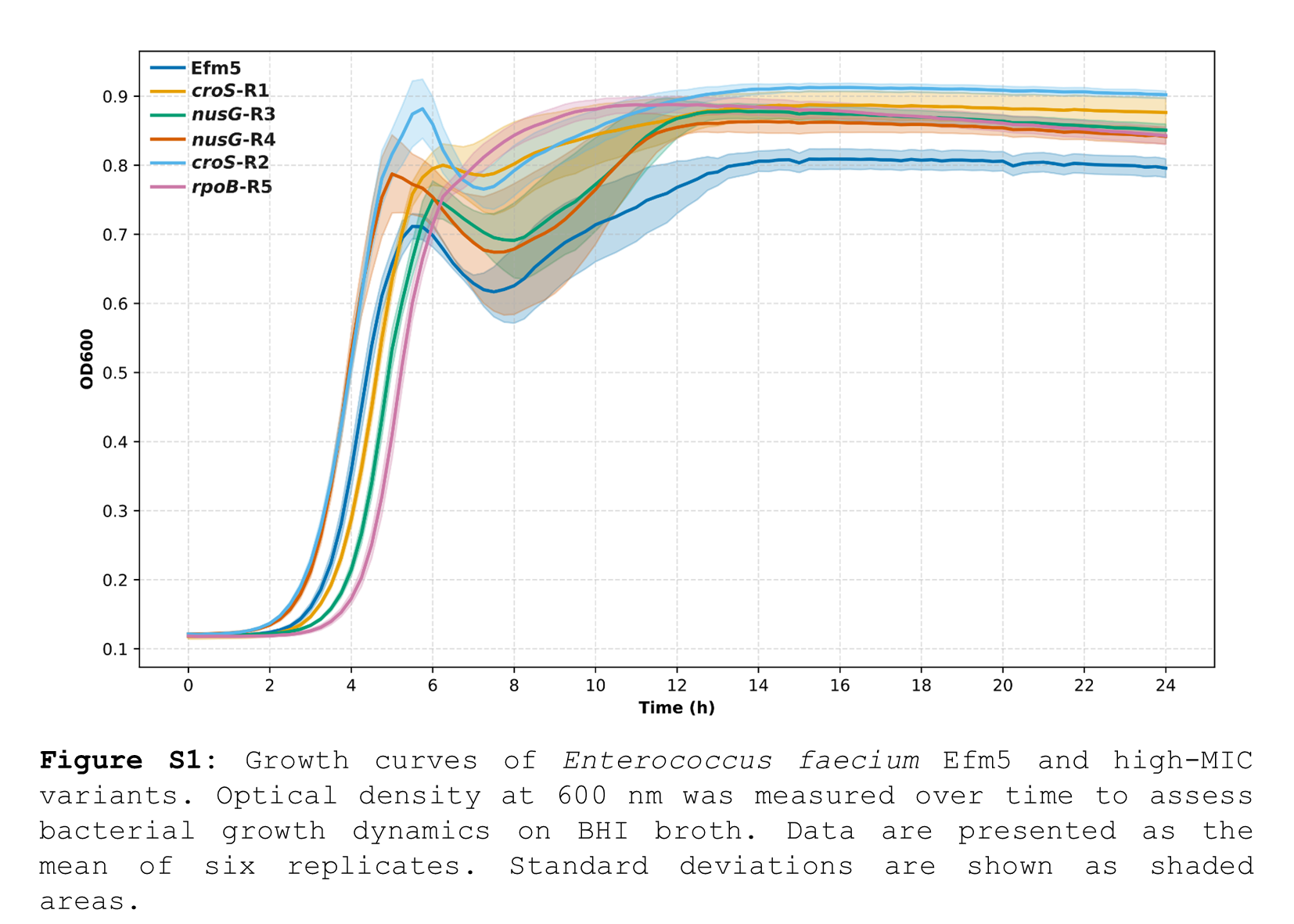

Supplement: Fig. S1 — Supplemental figure 1. [file aac.01948-25-s0001.tiff]

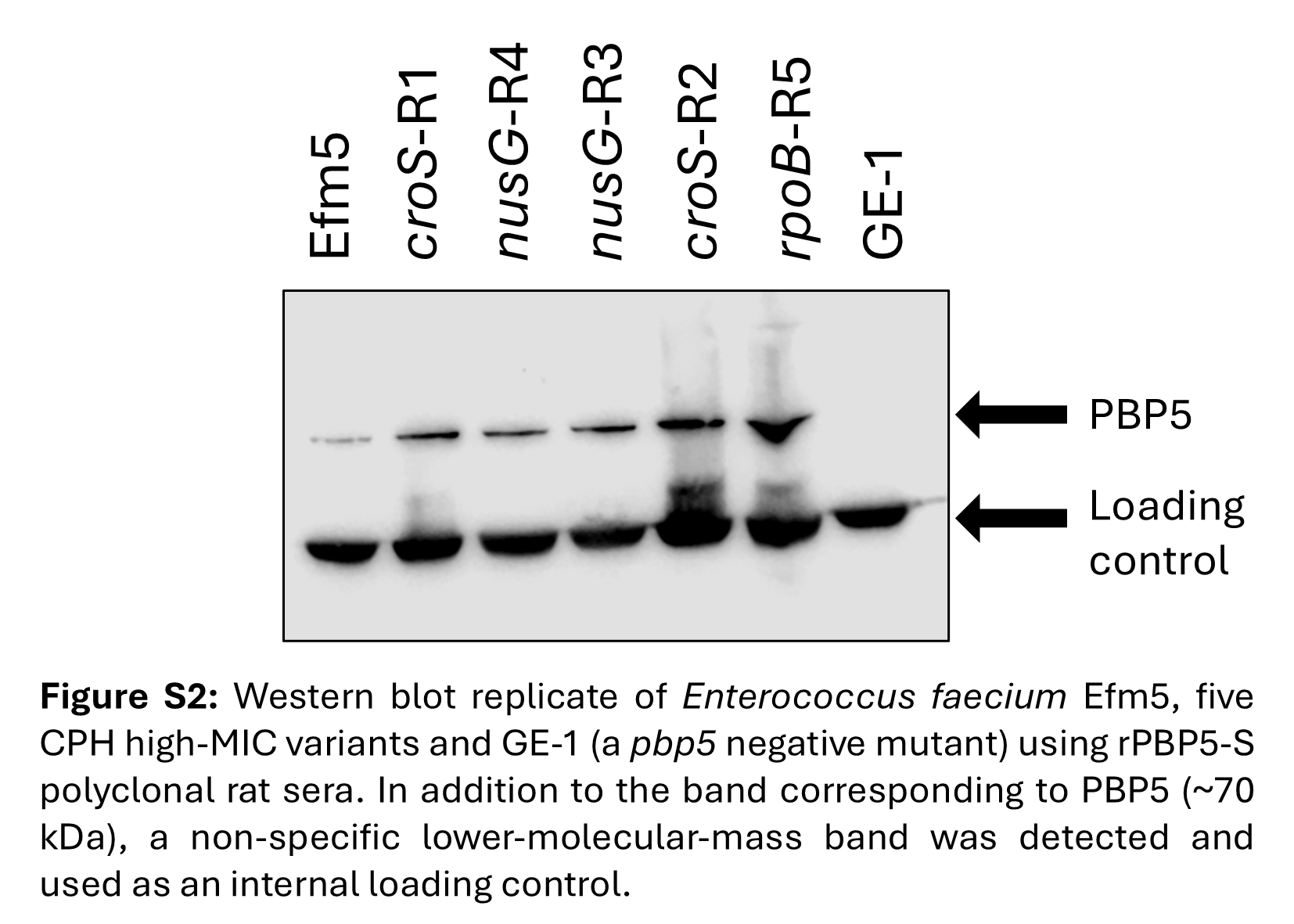

Supplement: Fig. S2 — Supplemental figure 2. [file aac.01948-25-s0002.tiff]

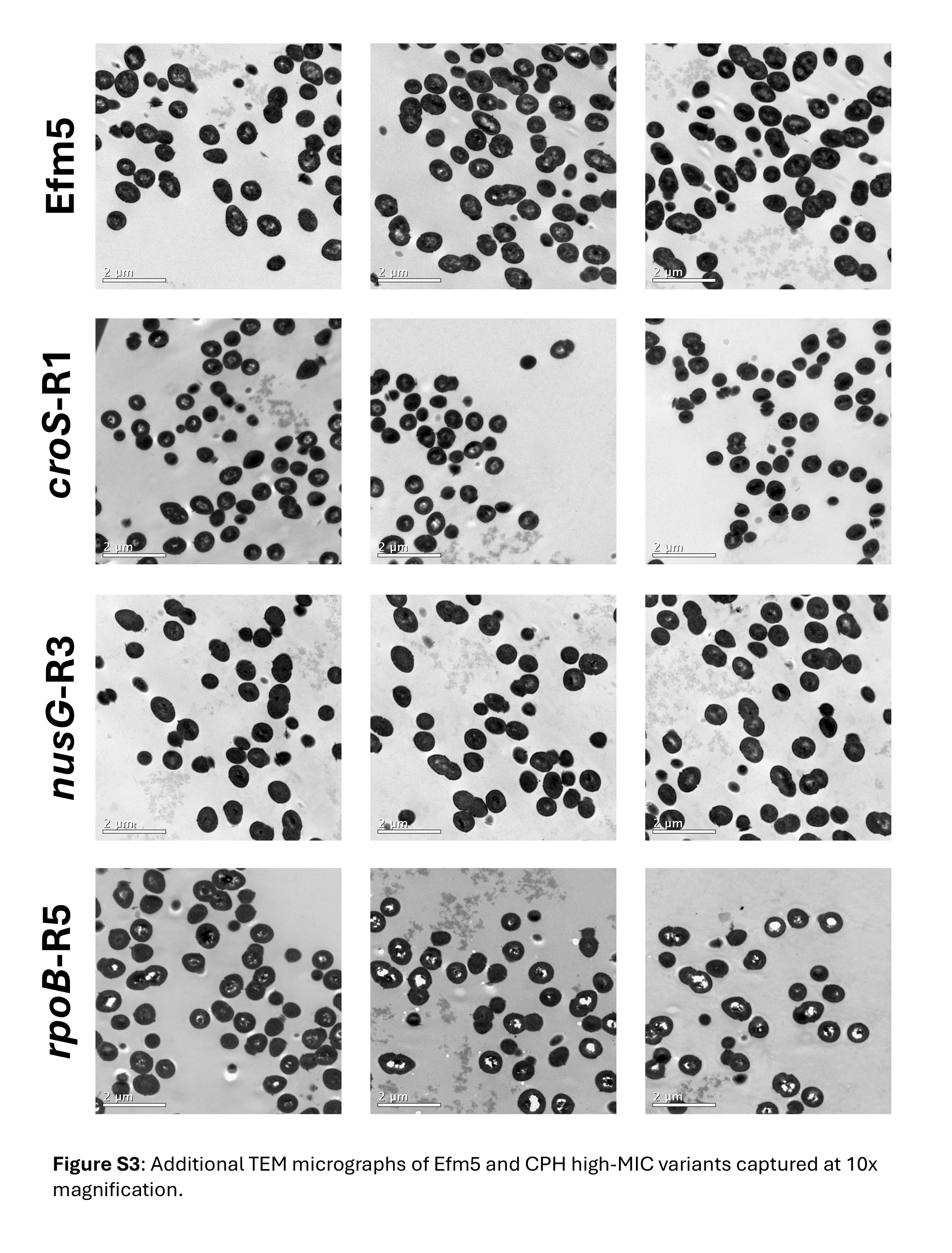

Supplement: Fig. S3 — Supplemental figure 3. [file aac.01948-25-s0003.tiff]

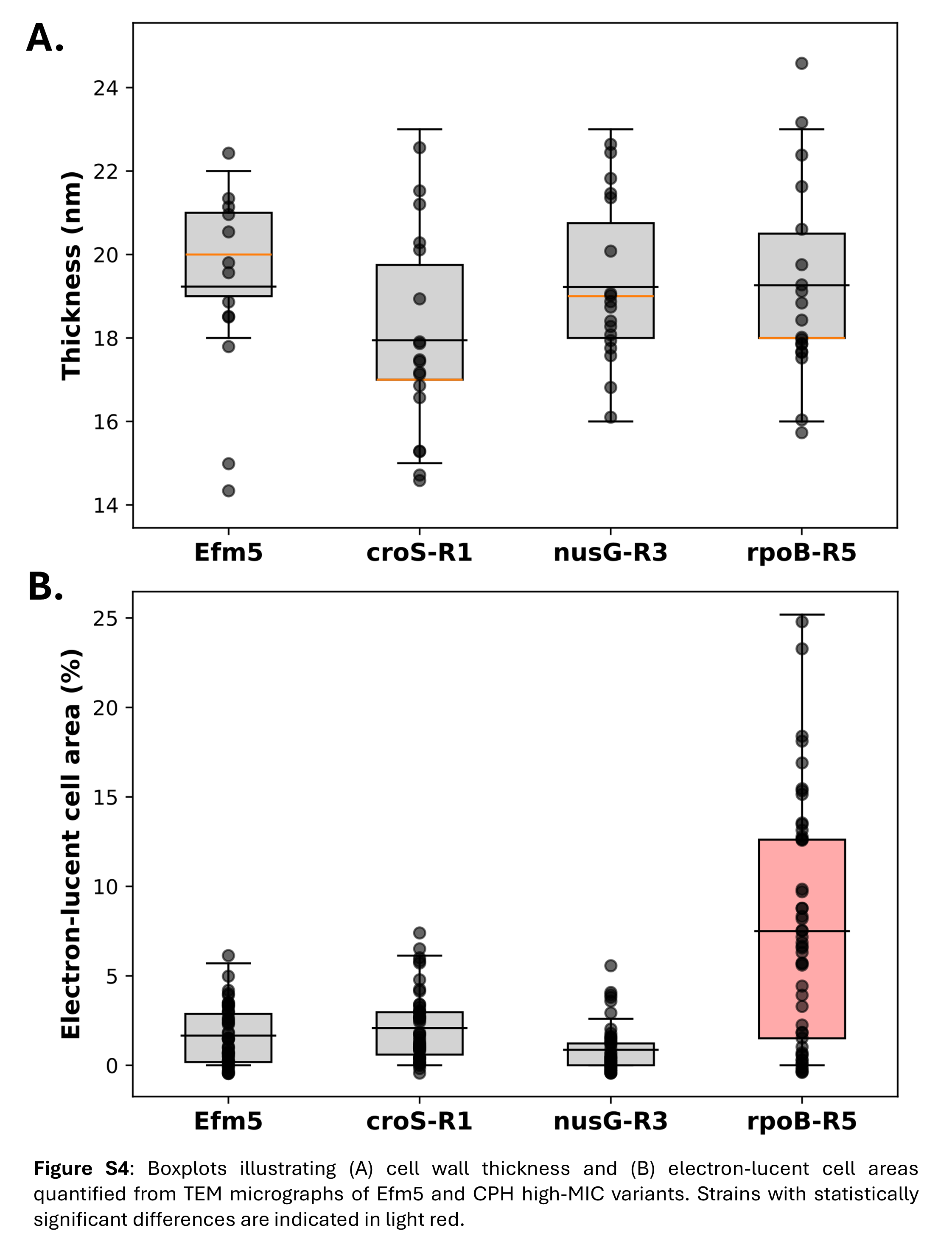

Supplement: Fig. S4 — Supplemental figure 4. [file aac.01948-25-s0004.tiff]

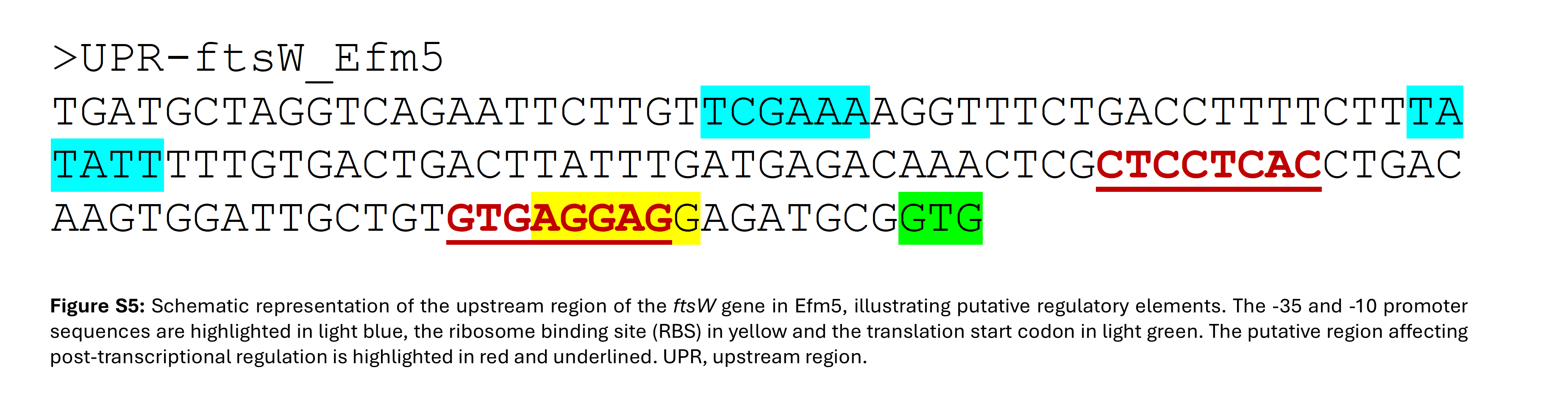

Supplement: Fig. S5 — Supplemental figure 5. [file aac.01948-25-s0005.tiff]
